# A case of bloodstream co-infection of *Saccharomyces cerevisiae* and *Candida glabrata* while using micafungin

**DOI:** 10.1186/s12879-023-08287-9

**Published:** 2023-05-16

**Authors:** Kento Furuya, Kenta Ito, Kyohei Sugiyama, Satoshi Tokuda, Hideyuki Kanemoto, Katsuhiko Kamei, Toshio Shimada

**Affiliations:** 1 Department of Clinical Laboratory Medicine, Shizuoka General Hospital, Kitaandou 4-27-1, Aoi-Ku, Shizuoka, Japan; 2 Department of Pharmacy, Shizuoka General Hospital, Shizuoka, Japan; 3 Department of Surgery, Shizuoka General Hospital, Shizuoka, Japan; 4 Medical Mycology Research Center, Chiba University, Chiba, Japan

**Keywords:** *Saccharomyces cerevisiae*, *Candida glabrata*, Fungemia, Fluconazole, Micafungin

## Abstract

**Background:**

*Saccharomyces cerevisiae* is ubiquitous in the gastrointestinal tract and known as brewer's or baker's yeast. We experienced a case of *S. cerevisiae* and *Candida glabrata* co-infectious bloodstream infection*.* It is rare to detect both *S. cerevisiae* and *Candida* species in blood cultures together.

**Case:**

We treated a 73-year-old man who developed a pancreaticoduodenal fistula infection after pancreaticoduodenectomy. The patient had a fever on postoperative day 59. We took blood cultures and detected *C. glabrata*. Thus, we started micafungin. On postoperative day 62, we retested blood cultures, and detected *S cerevisiae* and *C. glabrata*. We changed micafungin to liposomal amphotericin B. Blood cultures became negative on postoperative day 68. We changed liposomal amphotericin B to fosfluconazole and micafungin because of hypokalemia. He got well, and we terminated antifungal drugs 18 days after the blood cultures became negative.

**Conclusion:**

Co-infection with *S. cerevisiae* and *Candida* species is rare. In addition, in this case, *S. cerevisiae* developed from blood cultures during micafungin administration. Thus, micafungin may not be effective enough to treat *S. cerevisiae* fungemia, although echinocandin is considered one of the alternative therapy for *Saccharomyces* infections.

## Case presentation

**Fig. 1:**
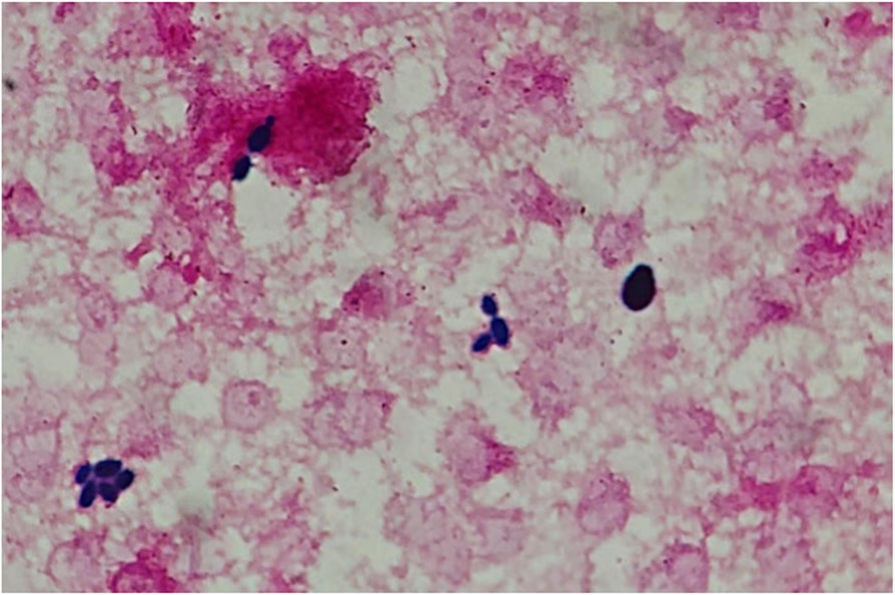
Gram stain of blood revealed two kinds of yeasts of different sizes. Later, we identified bigger one as *S. cerevisiae* and the other as *C. glabrata* (magnification × 1000)

A 73-year-old Japanese man received two courses of neoadjuvant therapy for pancreatic cancer. We used gemcitabine and cisplatin for neoadjuvant therapy. His pancreatic cancer stage was Stage IB after neoadjuvant therapy. After that, we performed a pancreaticoduodenectomy on him. Pathology showed adenocarcinoma with the invasion of the bile ducts and duodenum. On postoperative day 7, he had a fever. We diagnosed him with a pancreatic fistula infection and started piperacillin-tazobactam. However, blood cultures taken at that time were negative. We did not collect any other cultures. On postoperative day 39, he developed a fever again, and the blood culture showed *Enterococcus faecium*. We added vancomycin. We also added micafungin because he also had esophageal candidiasis. After the antibiotics change, his fever resolved quickly. On postoperative day 54, we terminated micafungin and changed antibiotics to ampicillin-sulbactam and vancomycin. On postoperative day 59, he had a fever-up again. We collected two blood cultures and changed antibiotics to piperacillin-tazobactam, vancomycin, and micafungin. *C. glabrata* was detected in one of two blood cultures, which appeared to be a single-size yeast on Gram stain. Minimal inhibitory concentration (MIC) for antifungal agents were as follows; micafungin ≤ 0.03 mg/L and fluconazole 16 mg/L. Thus, we continued micafungin 150 mg once a day. On postoperative day 62, we retested two blood cultures from veins at different sites to confirm that *C. glabrata* had disappeared from his blood. However, both blood cultures unexpectedly tested positive for two kinds of yeasts of different sizes on Gram stain (Fig. 1). We did not detect fungi in sputum or urine cultures. And we did not order other cultures, including his skin. We cultured yeasts with sheep blood agar (Nippon Becton Dickinson Co., Ltd., Tokyo, Japan) and Sabouraud’s dextrose agar (Nissui Pharmaceutical Co., Ltd., Tokyo, Japan). Matrix-assisted laser desorption/ionization time-of-flight-mass spectrometry (MALDI-TOF MS) (MALDI Biotyper, Bruker Japan, Ltd., Kanagawa, Japan) identified them as *S. cerevisiae* and *C. glabrata.* In addition, we asked the Medical Mycology Research Center, Chiba University, to perform a genetic analysis of the internal transcribed spacer region of the bigger yeast and demonstrated it was *S. cerevisiae*. The antifungal susceptibility test of the isolated *S. cerevisiae* was performed using the broth microdilution assay according to the Clinical and Laboratory Standards Institute approved standard M27 4th Edition. We also found *S. cerevisiae* and *C. glabrata* in his stool cultures.　Both were susceptible to amphotericin B (Table 1). Based on these results, we changed micafungin to liposomal amphotericin B 300 mg (5 mg/kg) once a day on postoperative day 64. Blood cultures were negative on postoperative day 68, but his serum potassium dropped to 2.1 mEq/L on postoperative day 73. His creatinine clearance was 53 mL/min, so it was difficult to determine the appropriate dose of fosfluconazole for *C. glabrata* fungemia according to the susceptibility test result. Thus, we changed liposomal amphotericin B to fosfluconazole 400 mg for *S. cerevisiae* and micafungin 150 mg for *C. glabrata* once a day. He got well, and then we stopped administering fosfluconazole and micafungin 18 days after the blood cultures became negative. After completing antifungal therapy, he had no recurrence of the fungal infection, and we discharged him.

**Table 1:** Susceptibility data on *S cerevisiae* and *C. glabrata*

	MIC	
Drugs	*S cerevisiae*	*C. glabrata*
amphotericin B	0.25	0.5
fluconazole	4	16
itraconazole	0.25	1
voriconazole	0.06	0.5
miconazole	0.06	1
micafungin	0.125	< = 0.03
caspofungin	0.5	0.5
flucytosine	< = 0.125	< = 0.125

## Discussion and conclusion

*S. cerevisiae* is known as brewer's or baker's yeast*.* And *S. cerevisiae* and *Candida* species are ubiquitous in the gastrointestinal tract [1]. However, co-infection with *S. cerevisiae* and *Candida* species was rare, occurring in only one of 530 candidemia cases [2]. Fortunately, we could notice the co-infection of *S. cerevisiae* and *C. glabrata* by careful observation of Gram stain in this case. In practice, although both appear as yeasts on Gram stain, they look different in size. As discussed below, risk factors for *S. cerevisiae* fungemia and candidemia are similar [3, 4]. And when we find different sizes of yeasts in the blood culture, we should utilize MALDI-TOF MS and genetic analysis to avoid overlooking rare fungal infections, including *S. cerevisiae*.

Risk factors of *Saccharomyces* fungemia and candidemia are similar. Common risk factors are using central venous catheters, tube feeding, history of broad-spectrum antibiotics use, and transplantation [3, 4]. As for *Saccharomyces* bloodstream infection, probiotics or supplements containing *Saccharomyces* are a risk factor, and 45.6% of *Saccharomyces* fungemia cases have used these probiotics [3, 5]. *Saccharomyces* containing probiotics and supplements are widely used in Europe and the United States of America because they may prevent *Clostridioides difficile* infection [5]. But this patient had never used them. In this case, the using broad-spectrum antibiotics matched the risk factors.

For *S. cerevisiae* infection, amphotericin B is the first-line drug, and azole or echinocandin is recommended as an alternative therapy [6]. One study showed that the success rate for treatment for *Saccharomyces* fungemia with amphotericin B was 85% (17/20 cases), and fluconazole was 80% (12/15 cases) [3]. In contrast, to our knowledge, four reports of echinocandin are used for *Saccharomyces* fungemia [7–10]. Three were successfully treated with echinocandin; one patient was treated with micafungin 100 mg/day [9] and the other with caspofungin [8, 10]. But the remaining one was unsuccessfully treated with caspofungin and successfully treated with liposomal amphotericin B, as in our case [7]. In our case, the treatment success rate for *Saccharomyces* fungemia with an echinocandin is 60% (3/5 cases). Thus, echinocandin monotherapy may not be effective for *S. cerevisiae* fungemia, even though *S. cerevisiae* and *C. glabrata* show similar susceptibility to antifungal drugs in vitro [10, 11]. In this case, 150 mg of micafungin was administered over one hour, and since liver function and body weight were not a problem, the maximum plasma concentration was estimated to be 12–16 mg/L [12]. The recommended dose of micafungin for severe and burn cases is 150–200 mg [12]. Therefore, the micafungin dose, in this case, was appropriate. One of the reasons for the breakthrough infection of *Saccharomyces* under micafungin administration was presumed to be the high MIC of *Saccharomyces* for micafungin. Further investigation may be necessary to determine the breakpoint of micafungin against *Saccharomyces*.　We initially selected liposomal amphotericin B for nine days and switched to fosfluconazole because the patient developed marked hypokalemia. The optimal dose of fluconazole for *Saccharomyces* infection is not known. Fluconazole doses varied from 100–400 mg/day in adult cases of *Saccharomyces* fungemia, but the most used dose was 400 mg/day [9, 13–16], and we also treated with fosfluconazole 400 mg/day.

In conclusion, during micafungin use, we experienced a case of *S. cerevisiae* and *C. glabrata* co-infectious bloodstream infection. Risk factors for *Saccharomyces* fungemia and candidemia are similar. Therefore, when we diagnose candidemia and notice different sizes of yeast on Gram stain, we need to pay attention not to miss the co-infection of fungi, including *S. cerevisiae*. Although echinocandin is recommended as an alternative therapy for amphotericin B for *Saccharomyces* infection, it may be better to choose azole than echinocandin if amphotericin B is not available. Furthermore, since *C. glabrata* is intrinsically resistant to azoles, combining azoles and echinocandin may be necessary for cases co-infected with *C. glabrata* and *S. cerevisiae* when amphotericin B cannot be used.

## Data Availability

Not applicable.
